# A brief harvesting-freezing delay significantly alters the kidney metabolome and leads to false positive and negative results

**DOI:** 10.1152/ajprenal.00131.2024

**Published:** 2024-08-29

**Authors:** Yahya Alsawaf, Igor Maksimovic, Jamie Zheng, Song Zhang, Ivan Vuckovic, Petras Dzeja, Slobodan Macura, Maria V. Irazabal

**Affiliations:** ^1^Mayo Translational PKD Center, Division of Nephrology and Hypertension, Mayo Clinic, Rochester, Minnesota, United States; ^2^Metabolomics Core, Mayo Clinic, Rochester, Minnesota, United States; ^3^Department of Cardiovascular Medicine, Mayo Clinic, Rochester, Minnesota, United States; ^4^Department of Biochemistry, Mayo Clinic, Rochester, Minnesota, United States

**Keywords:** ischemia, kidney, metabolomics, organ harvesting, PKD, TCA cycle

## Abstract

Abnormalities in distinct metabolic pathways have been associated with the pathogenesis and progression of many forms of kidney disease. Metabolomics analyses can be used to determine organ-specific metabolic fingerprints and, ideally, should represent the metabolic state of the organ at the exact moment the sample is harvested. However, conventional harvesting methods depend on posteuthanasia tissue harvest, which results in ischemia conditions and metabolome changes that could potentially introduce artifacts into the final studies. We recently optimized a modified clamp-freezing technique for rodent kidney harvesting and freezing, significantly reducing ischemia and freezing times and granting a closer snapshot of in vivo metabolism. In this study, we characterized and compared the metabolome of kidneys harvested using our modified approach versus traditional techniques to determine which metabolites are preferentially affected by a brief lapse of ischemia and freezing delay and which are more stable. We used Sprague-Dawley rats as a model of wild-type (WT) kidneys and PCK [polycystic kidney disease (PKD)] rats as a model of chronic kidney disease kidneys. Finally, we compared the metabolic profile of clamp-frozen and delayed WT and PKD kidneys to determine which metabolic changes are most likely observed in vivo in PKD and which could be subjected to false positive or negative results. Our data indicate that a short harvesting-freezing delay is sufficient to impart profound metabolic changes in WT and PKD kidneys, leading to false positive and negative differences when comparing these genotypes. In addition, we identified a group of metabolites that were more stable. Interestingly, while the delay had a similar effect between WT and PKD, there were notable differences. The data obtained indicate that the quick clamp-freezing technique for kidney metabolomics provides a more accurate interpretation of the in vivo metabolic changes associated with the disease state.

**NEW & NOTEWORTHY** Our study shows that a brief harvesting-freezing delay associated with organ collection and freezing can significantly alter the kidney metabolic profile of both male and female wild-type and a genetic model of chronic kidney disease. Importantly, given that the effect of this delay differs among genotypes, it is not safe to assume that equally delaying harvesting-freezing in wild-type and polycystic kidney disease kidneys adequately controls this effect, ultimately leading to false positive and negative results among different renal diseases.

## INTRODUCTION

The mammalian kidney is continuously active with marked intrinsic metabolic activity and high energy demands, consuming the second-highest amount of oxygen per gram of tissue (2.7 mmol/kg/min) (1). Moreover, by glomerular filtration, tubular secretion, or net production, kidneys can directly control circulating metabolite levels and impact systemic metabolism and their complicated metabolic activities. Therefore, renal abnormalities can be caused and sustained by a vicious cycle that is triggered by disturbances in the metabolism of the kidneys and the blood. Not surprisingly, more and more evidence suggests that abnormalities in distinct metabolic pathways are implicated in the pathogenesis and progression of many forms of chronic kidney disease (CKD), including polycystic kidney disease (PKD) (2–7).

A relatively recent omics technology called “metabolomics” determines the concentration of many metabolites in a specific solid tissue or biofluid from a living entity (8). NMR has extensively been used for monitoring metabolic changes with the main advantage of requiring little sample preparation and high analytical reproducibility. In addition, it is a nondestructive technology and has less inter-laboratory variation. Typically, NMR spectroscopy allows for the simultaneous quantification of 20–50 metabolites (9). The main disadvantage of NMR is the lower sensitivity, therefore requiring a relatively large amount of sample compared to mass spectrometry, with metabolite concentrations being the limiting factor. While biofluids effectively integrate the metabolic changes in a living organism, tissue samples can be used to determine organ-specific metabolic fingerprints. In addition, when correctly performed, tissue metabolomics can reveal a snapshot of the tissue in vivo metabolism, potentially identifying underlying mechanisms associated with the disease. However, intracellular metabolite compositions can quickly shift during sample collection and preparation, changing the analytical readout and, subsequently, in vivo metabolic state conclusions. To reduce artifacts that complicate biological interpretation, sample collection and preparation must be done adequately to yield accurate and consistent results.

Metabolic reprogramming favoring aerobic glycolysis, glutamine dependence, and defects in fatty acid oxidation are among the most frequently observed changes associated with kidney disease, including in PKD (7, 10–22). Still, even for the same disease, findings sometimes differ among studies, likely due to differences in study design, animal models, or disease stage. More importantly, studies do not report controlling for critical factors influencing the outcome, such as the effect of harvesting and freezing techniques for halting tissue metabolic activities. This might result in a critical problem given that many glycolytic metabolites have turnover times of a few seconds (<30 s) with rapid changes in response to ischemia (23, 24). Furthermore, the time it takes to stop enzymatic activity by freezing depends on the sample size and can further impact metabolite concentrations. Thus minimization of ischemia time during organ harvesting, followed by rapid termination of enzyme activity, is essential to achieve an accurate readout of the in vivo kidney metabolome.

We recently optimized a protocol for rodent kidney harvesting, freezing, and sample preparation for metabolomics analyses and showed temporal changes in adenylates (ATP, ADP, and AMP) and lactate on kidney tissue in response to ischemia and freezing delay (25). We introduced a modified clamp-freezing technique, where a rapidly excised kidney is pressed thin and frozen between aluminum blocks, reducing ischemia and freezing times to 7 and 5 s, respectively, granting a closer snapshot of in vivo metabolism.

In the present study, we extended our prior analysis and performed a comprehensive metabolomic characterization of the left kidney harvested following our modified clamp-freezing technique and the right kidney using traditional harvesting and freezing techniques (delayed) in Sprague-Dawley (SD) rats as a model of wild-type (WT) kidneys and PCK (PKD) rats as a model of CKD. Finally, we compared the metabolic profile of the clamp-frozen and delayed WT and PKD kidneys to better approximate the in vivo changes observed in PKD. Our results indicate that a short harvesting-freezing (HF) delay is sufficient to result in profound changes in the metabolic profile of WT and PKD kidneys, leading to false positive and negative results when comparing genotypes. On the other hand, we identified a group of metabolites that were more stable and might be more reliable, independent of the harvesting and freezing approach. The most significant changes in WT and PKD kidneys were related to purine metabolism. However, some notable differences between WT and PKD kidneys were observed with ischemia, likely reflecting underlying metabolic abnormalities previously reported in PKD. Therefore, our observations support clamp-freezing techniques for kidney metabolomics for a more accurate interpretation of the in vivo metabolic changes underlying the disease state.

## MATERIALS AND METHODS

### Study Design, Experimental Animals, and Organ Harvesting-Freezing Approaches

The study was conducted under the A28715 Institutional Animal Care and Use Committee (IACUC) protocol. The Mayo Clinic IACUC approved the experimental protocol, and all methods were carried out following the National Institutes of Health, United States Department of Agriculture, and American Association for Laboratory Animal Science regulations. All methods are reported in accordance with Animal Research: Reporting of In Vivo Experiments guidelines. Both male and female SD rats (RRID:RGD_70508) were used as models for WT kidneys, and male and female PCK rats were used as models of chronic kidney disease kidneys (PKD). The PCK rat utilized in this study is an advantageous PKD model that, although the pattern of inheritance is autosomal recessive, it resembles many of the phenotypic characteristics of human ADPKD and has been successfully used in many preclinical trials (26–31). The SD rat is the background for our PCK model and hence constitutes the best WT control animals. Animals were generated at the Mayo Translational PKD Center. Twenty SD (*n* = 10 males and *n* = 10 females) and 20 PCK (*n* = 10 males and *n* = 10 females) rats of 29–47 days of age were included in the study.

A 24-h urine sample was collected using metabolic cages before animal euthanasia, and a blood sample was collected at the time of euthanasia for a biochemical profile. No animals were lost during the study, and all were available for analysis. Experimenters were not blinded to the animal’s genotype because PCK rat kidneys were visibly different from controls.

Two different organ harvesting and freezing approaches were performed for the left and right kidneys to evaluate the preservation of sensitive metabolite levels. Briefly, the left kidney was harvested and frozen using a modified clamp-freezing technique following our optimized protocol (25), while the right kidney was harvested after animal exsanguination through a cardiac puncture and frozen in liquid N_2_ using standard snap freezing techniques. Kidney tissue in both methods was pulverized in a mortar cooled with liquid N_2_ and kept at −80°C until sample preparation. Whole body and kidney weights were obtained at the time of euthanasia. Ischemia time was determined using a chronometer and defined as the time between vessels’ clamping and the kidney’s placement within the aluminum mortar or the time between cardiac puncture and the kidney’s submersion in liquid N_2_ for the left and right kidneys, respectively. Freezing time was estimated according to the theory of temperature conduction in flat-pressed tissue using the equation previously described (32–34). The total time for each kidney was determined by adding ischemia and freezing times.

### Tissue Sample Preparation

Tissue sample preparation was performed similarly for the left and right kidneys. A 30-mg aliquot of the frozen powder was homogenized on ice-cooled 6% HClO_4_, vortexed for 5 s, and placed on ice for 10 min. Next, the sample was centrifuged for 10 min at 10,000 *g* at 4°C, and the supernatant was transferred to an ice-cooled EP tube. The supernatant was neutralized to pH 7.0 with ice-cooled 2 M KHCO_3_ and left on ice for 10 min. Samples were next centrifuged for 10 min at 10,000 *g* at 4°C to precipitate the potassium perchlorate salt, and the supernatant (400 μL) was transferred to another ice-cooled EP tube. Then, 100 μL of 0.1 M phosphate buffer and 50 μL of 1 mM TSP-*d*_4_ solution in D_2_O were added to each EP tube. Tubes were vortexed for 10 s and then centrifuged at 10,000 *g* for 15 min. The supernatant (550 μL) was transferred to 5-mm NMR tubes.

### NMR Data Acquisition

The spectra were acquired on a Bruker 600-MHz instrument equipped with a BBI probe head, using a one-dimensional NOESY pulse sequence with presaturation (noesygppr1d) and a 90° pulse (∼13 μs), 4.68-s acquisition time, and 4-s relaxation delay. The temperature was 298 K. All spectra were acquired with 64 k data points and 8,417 Hz (14 ppm) spectral width. The 128 scans were applied for each sample (total acquisition time: 18.5 min).

### NMR Data Processing

The recorded ^1^H-NMR spectra were phase and baseline-corrected using TopSpin 3.5 software (RRID:SCR_014227). The ^1^H-NMR spectra were transferred to a workstation, binned into 0.04 ppm integral regions over a chemical shift range of 0.7–9.34 ppm, and the regions within each bin were integrated using Chenomx NMR Suite 8.1 software. The region containing the water (4.68–5.71 ppm) resonance was removed from the analysis for all groups. The ^1^H-NMR spectra were normalized to the total sum of the spectral integrals (TA) and to the internal standard (TSP) to compensate for differences in sample concentration, and multivariate analyses were performed as described below.

For quantification of individual metabolites, the processed spectra were analyzed using Chenomx NMR Suite 8.1 software, and compounds were identified by comparing to the database Chenomx 600 MHz Version 10 and quantified based on internal standard (TSP-*d*_4_) peak integral. The concentrations of identified metabolites were exported as milligrams per deciliter in the NMR sample and then recalculated as milligrams per gram of wet tissue.

### Statistical and Pathway Analysis

Animal and laboratory characteristics were analyzed using PRISM9 (GraphPad Software, La Jolla, CA, RRID:SCR_002798). The Shapiro-Wilk test was used to determine deviation from normality. Data normally distributed are expressed as mean ± SD, whereas data nonfollowing a Gaussian distribution are expressed as median (minimum-maximum). The paired (left vs. right kidneys) and unpaired (WT vs. PKD) Student’s *t* test or nonparametric tests (Wilcoxon signed-rank or Mann-Whitney *U* test) were performed according to the study design. All tests were two-sided with an α level of 0.05.

The statistical analysis of the normalized (TA and TSP) binned NMR spectra was conducted using MetaboAnalyst v.5.0 (University of Alberta, RRID:SCR_015539) after performing autoscaling, which uses each variable’s standard deviation as the scaling factor (35). Multivariate analysis included principal component analysis (PCA) and partial least squares discriminant analysis (PLS-DA). PCA was used to identify any innate trends and potential outliers within the data. PLS-DA was used to obtain additional information among each genotype, including differences in the metabolite composition of groups and variable importance on projection (VIP) values. The results were visualized as score plots to show the group clusters, and the VIP scores were calculated to identify the spectral regions most important for differentiating between the two harvesting and freezing approaches for each genotype. The model’s outcomes were assessed using *Q*^2^ (the predictive ability of the model) and *R*^2^ (goodness of fit) after leave-one-out cross-validation (LOOCV), and models were accepted as valid if *Q*^2^ >0.5. The PLS-DA model’s statistical significance was tested based on the permutation test with respect to separation distance (B/W, 2,000 permutations).

Quantitative uni- and multivariate metabolomics analyses were performed using MetaboAnalyst 5.0. Metabolite concentrations normalized by tissue weight were auto-scaled, and paired or unpaired Student’s *t* tests were applied, with a Benjamini-Hochberg false discovery rate (FDR) post hoc correction to correct for multiple comparisons (FDR < 0.05 for statistical significance). Pathway analysis was performed using MetaboAnalyst 5.0, using metabolic set enrichment analysis (MSEA). Pathways were considered significantly enriched if *P* < 0.05, impact > 01, and the number of metabolites hits in the pathway > 1.

## RESULTS

### Animal Characteristics and Ischemia-Freezing Times in WT and PKD

The main animal characteristics and ischemia-freezing times are presented in Table 1, and representative abdominal MRI images of WT and PKD animals are shown in Supplemental Fig. S1. Sex-specific comparisons are reported in Supplemental Table S1. Age and body weight were not different between WT and PKD, and there were no sex differences for either genotype. However, as expected, left and right kidney weights were higher in PKD versus WT for both sexes. Similarly, total kidney weight and total kidney weight adjusted by body weight were higher in PKD compared to WT, but there were no sex differences at this early stage. Right ischemia time was higher than left ischemia time by design, but left and right ischemia times did not differ between WT and PKD for males or females. Flattened left kidneys were not different between WT and PKD, resulting in similar freezing and total times. However, PKD right kidneys were thicker than WT right kidneys, resulting in higher freezing times for males and females, although no difference between sexes was found. Despite this, the total time for the right kidneys was not different than in WT animals for males or females. Renal function parameters were not different between WT and PKD.

**Table 1.** Animal characteristics

Strain	SD	PCK	SD Versus PCK
Number, male/female	10/10	10/10	
Age, days	38.0 (30.0–47.0)	38.5 (29.0–47.0)	0.9552
BW, g	148.5 (102.0–266.0)	149.0 (100.0–252.0)	0.6636
LKW, g	0.9 (0.5–1.3)	1.3 (0.7–1.9)	**0.0152**
RKW, g	0.9 (0.5–1.2)	1.3 (0.6–1.9)	**0.0198**
TKW, g	1.8 (1.0–2.4)	2.6 (1.4–3.8)	**0.0137**
TKW/BW, %	1.1 ± 0.2	1.6 ± 0.2	**<0.0001**
LK ischemia time, s	6.9 ± 1.1	6.5 ± 1.1	0.2615
LK thickness, mm	2.0 ± 0.2	2.1 ± 0.3	0.1505
LK freezing time, s	4.6 ± 1.0	5.3 ± 1.6	0.1043
LK time, s	11.5 ± 1.5	11.8 ± 1.7	0.5528
RK ischemia time, s	395.0 ± 28.3	381.8 ± 24.0	0.1215
RK thickness, mm	7.3 ± 1.3	8.9 ± 1.1	**<0.0001**
RK freezing time, s	65.2 ± 21.6	95.3 ± 22.7	**<0.0001**
RK time, s	460.1 (396.8–494.5)	477.1 (423.7–548.2)	0.1716
BUN, mg/dL	14.4 ± 1.5	14.2 ± 1.4	0.7410
Creatine, mg/dL	0.4 ± 0.0	0.4 ± 0.0	0.2557
24-h urine, mL	7.9 ± 3.4	7.6 ± 3.2	0.7918

BUN, blood urea nitrogen; BW, body weight; LK, left kidney; LKW, left kidney weight; RK, right kidney; RKW, right kidney weight; SD, Sprague-Dawley; TKW, total kidney weight. LK time and RK time represent the time between the end of the blood supply and the complete freezing of the organ. Bold font indicates statistical signficance.

### Brief Harvesting-Freezing Delay Resulted in WT and PKD Kidney Metabolic Changes

**Figure 1.**
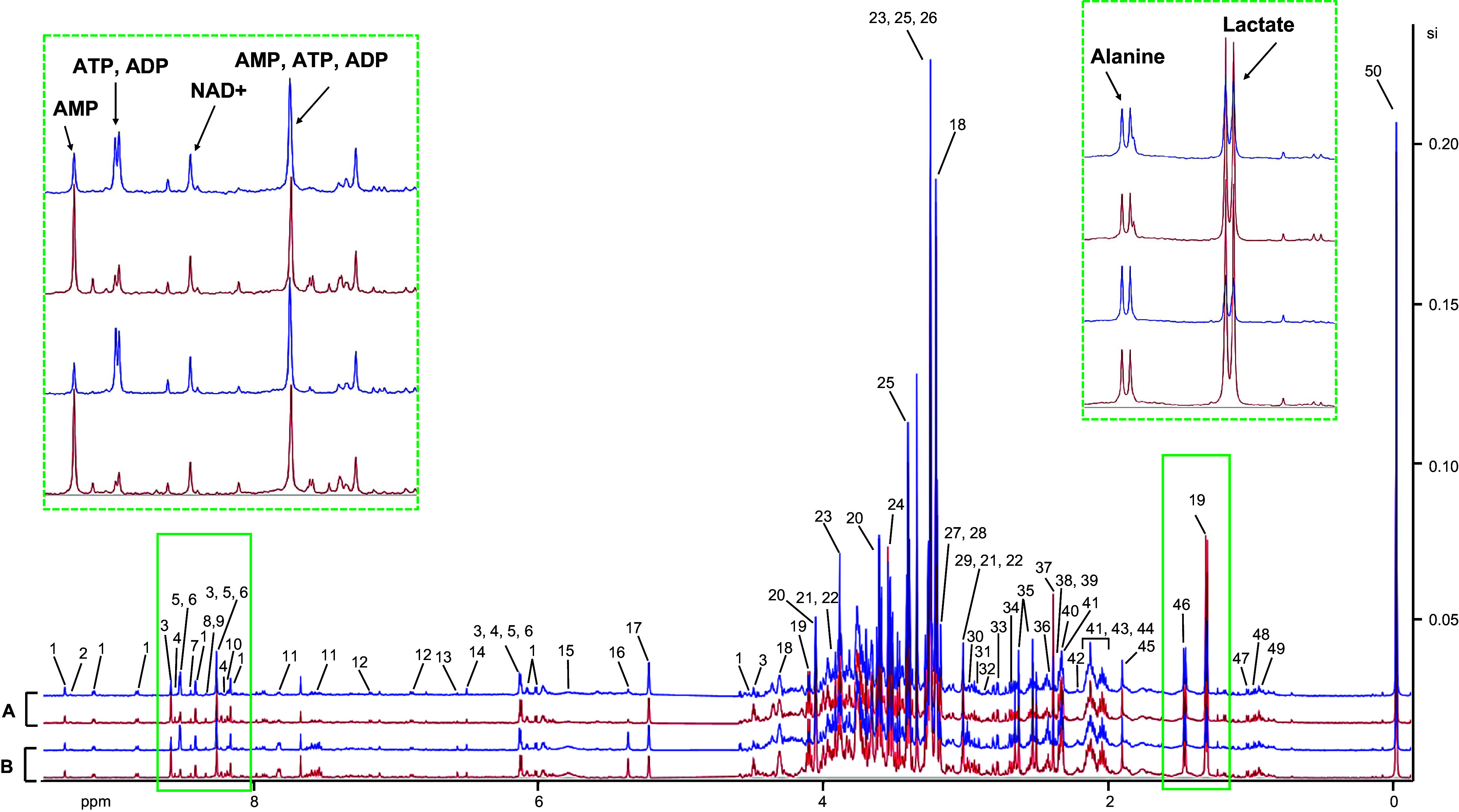
Representative one-dimensional ^1^H-NMR spectra of the kidney tissue extracts. *A* and *B*: 600-MHz ^1^H-NMR spectra of kidney extract samples from the left (blue) and right (red) wild-type (*A*) and polycystic kidney disease (*B*) animals. Green dashed boxes represent the green boxed areas at higher magnification. *1*: NAD^+^; *2*: NADP^+^; *3*: AMP; *4*: IMP; *5*: ATP; *6*: ADP; *7*: formate; *8*: inosine; *9*: adenosine; *10*: hypoxanthine; *11*: hippurate; *12*: tyrosine; *13*: *trans*-aconitate; *14*: fumarate; *15*: urea; *16*: allantoin; *17*: glucose; *18*: *sn*-glycero-3-phosphocholine; *19*: lactate; *20*: *myo*-inositol; *21*: creatinine phosphate; *22*: creatine; *23*: betaine; *24*: glycine; *25*: taurine; *26*: trimethylamine *N*-oxide; *27*: *O*-phosphocholine; *28*: choline; *29*: creatinine; *30*: α-ketoglutarate; *31*: asparagine; *32*: *N,N*-dimethylglycine; *33*: aspartate; *34*: dimethylamine; *35*: citrate; *36*: glutamine; *37*: succinate; *38*: malate; *39*: pyruvate; *40*: proline; *41*: glutamate; *42*: acetone; *43*: methionine; *44*: glutathione; *45*: acetate; *46*: alanine; *47*: valine; *48*: isoleucine; *49*: leucine; and *50*: TSP (internal standard).

Representative one-dimensional ^1^H-NMR spectra of the kidney tissue extracts from the left and right WT and PKD kidneys are shown in Fig. 1. Compounds were identified by comparing to the database Chenomx 600 MHz version 10 and supported by literature data. Global trends in spectral peak patterns can be quickly and reliably identified via spectral binning, eliminating the requirement for metabolite identification beforehand. Despite requiring human selection of the integration zones, spectral binning is a relatively simple technique for extracting global ^1^H-NMR signals. Therefore, we first proceeded with binning analysis to identify global differences between approaches.

**Figure 2.**
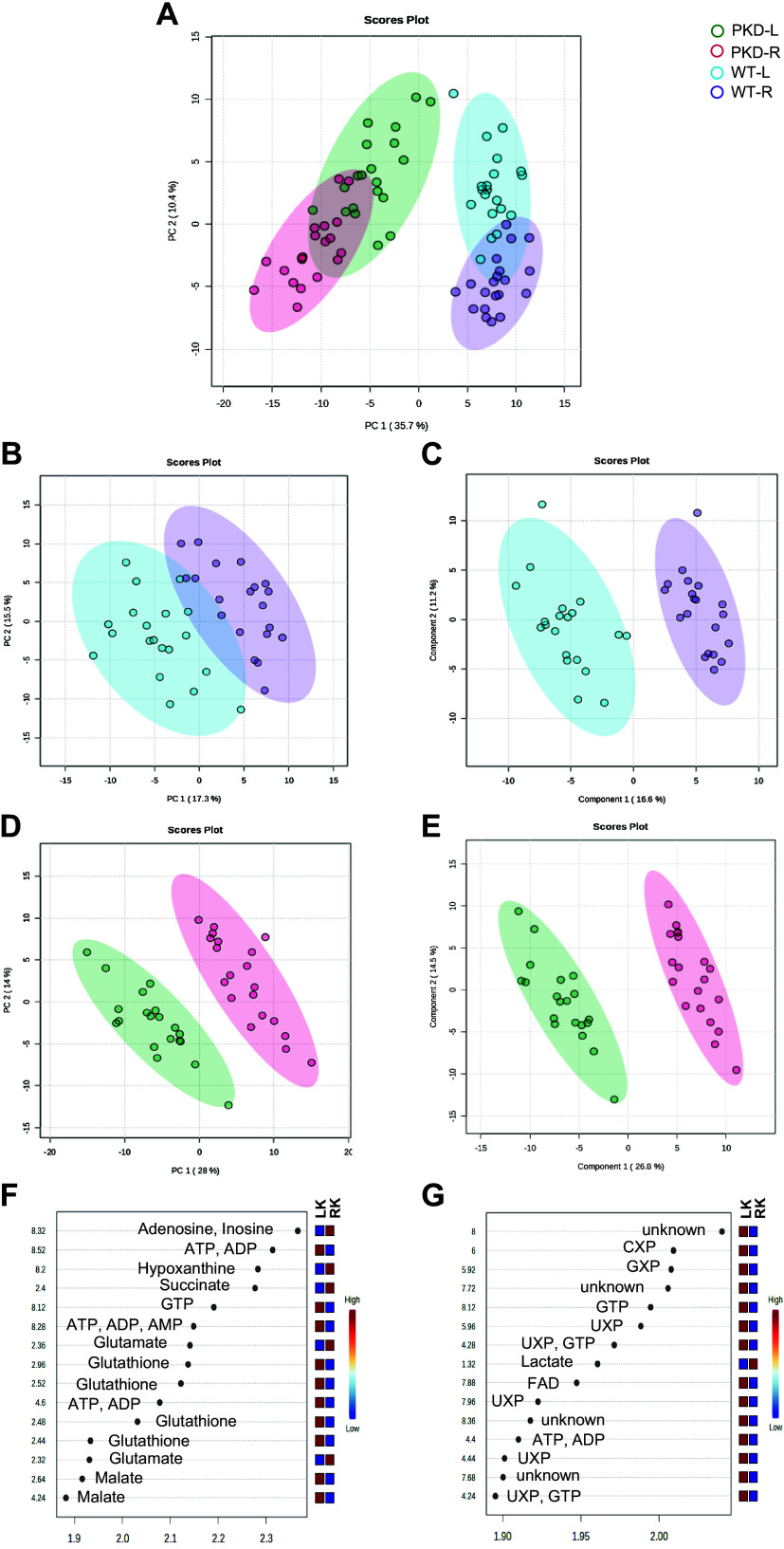
Proton nuclear magnetic resonance (^1^H-NMR) spectra analysis normalized to total area (TA) based on two different harvesting and freezing approaches in wild-type (WT) and polycystic kidney disease (PKD) kidneys. *A*: unsupervised principal component (PC) analysis score plot based on binned spectra normalized by TA, discriminating between different genotypes (WT and PKD) and approaches [left (L) and right (R) kidneys (LK and RK) from the same animal]. *B–E*: PC analysis (*B* and *D*) and partial least squares discriminant analysis (*C* and *E*) score plots, discriminating between LK and RK from the same animal, in WT (*B* and *C*) and PKD (*D* and *E*). *F* and *G*: variable importance on projection scores and metabolites related to bins with different concentrations among the LK and RK for WT (*F*) and PKD (*G*) (blue is low and red is high relative concentration). The explained variances along the PCs are shown in brackets. Shaded areas represent the 95% confidence regions. ADP, adenosine diphosphate; AMP, adenosine monophosphate; ATP, adenosine triphosphate; CXP, cytidine mono-, di-, and triphosphate; FAD, flavin adenine dinucleotide; GTP, guanosine triphosphate; GXP, guanosine mono-, di-, and triphosphate; UXP, uridine mono-, di-, and triphosphate.

The unsupervised PCA of the binned ^1^H-NMR spectra normalized to TA showed good separation between the harvestings and freezing approaches in the WT and PKD kidneys (Fig. 2*A*). A clear separation along PC1 was observed between the different genotypes, explaining 35.7% of the total variance within the data. A good separation while maintaining partial overlap was observed between the different approaches in the WT and PKD kidneys along PC2, which explained 10.4% of the total variance. These results indicate that the kidney tissue metabolome differed between genotypes and the approaches for both genotypes. No extreme outliers were detected; all samples were included in further analysis.

Next, we evaluated the different harvesting and freezing approaches for the left and right WT kidneys. The unsupervised PCA showed good separation between the approaches with minimal overlap along PC1, explaining 17.3% of the total variance within the data (Fig. 2*B*). We proceeded with PLS-DA, and the resulting score plots of *component 1* versus *component 2* showing a clear separation is presented in Fig. 2*C*. Comparison between the two approaches in WT kidneys led to a model with good predictive values (*Q*^2^ = 0.8717 and *R*^2^ = 0.9264), which was further validated based on the permutation test with respect to the separation distance (*P* < 0.001). The computed VIP scores and overall coefficient scores from the PLS-DA for each bin are shown in Supplemental Table S2, and the top 15 features are shown in Fig. 2*F*. We next assigned metabolites to the top 15 spectral bins based on their chemical shifts using literature data. The chemical shifts that most contributed to differentiating the two approaches in the WT kidneys were attributed to adenosine, inosine, ATP, ADP, hypoxanthine, succinate, GTP, AMP, glutamate, glutathione, and malate.

In the case of the PKD kidneys, the unsupervised PCA showed an excellent separation between the approaches along PC1, explaining 28% of the total variance within the data (Fig. 2*D*). PLS-DA score plots for the PKD kidneys are shown in Fig. 2*E*. Similarly to WT, comparing the two approaches in PKD kidneys led to a model with good predictive values (*Q*^2^ = 0.9428 and *R*^2^ = 0.9666), which was further validated based on the permutation test with respect to the separation distance (*P* < 0.001). The computed VIP scores and overall coefficient scores from the PLS-DA for each bin are shown in Supplemental Table S3, and the top 15 features are shown in Fig. 2*G*. In the PKD kidney, chemical shifts that most contributed to differentiating the two approaches were attributed to CXP, GXP, GTP, UXP, lactate, FAD, ATP, and ADP. Notably, the spectral bin that was the model’s most significant component was an unidentified metabolite at around 8 ppm. Analyses using TSP-normalized spectra resulted in similar findings in both groups (Supplemental Fig. S2 and Supplemental Tables S4 and S5).

Finally, we investigated if there were sex-specific differences and their relationship with the genotype and approach. Males and females overlapped in the PCA score plot in both genotypes and approaches (Supplemental Fig. S3*A*), indicating high similarity between both sexes. We next focused on the WT animals. Although males and females separated using PLS-DA (Supplemental Fig. S3, *B* and *C*) in each approach, the resulting models were not significant and were therefore not accepted (*P* = 0.512 and *P* = 0.308, respectively). Similarly to WT, although PKD males and females separated using PLS-DA (Supplemental Fig. S3, *D* and *E*) in each approach, the resulting models were not significant and were therefore not accepted (*P* = 0.2305 and *P* = 0.308, respectively).

### Harvesting-Freezing Delay Changes Are Consistent With Those Caused by Ischemic Anoxia in the WT Kidney

While spectral binning is computationally advantageous and less laborious, peak overlap often prevents a one-to-one mapping between integration regions and metabolites, even with good spectral alignment. Therefore, one limitation of the simplicity of spectral binning is the limited biological interpretability of the resulting features. Metabolite profiling on the other hand, although it is more laborious and time consuming, allows the identification and quantification of metabolites through fitting the sample spectrum to purely known metabolite spectra. To obtain a better biological interpretation of the data, we proceed with metabolites profiling analysis.

**Figure 3.**
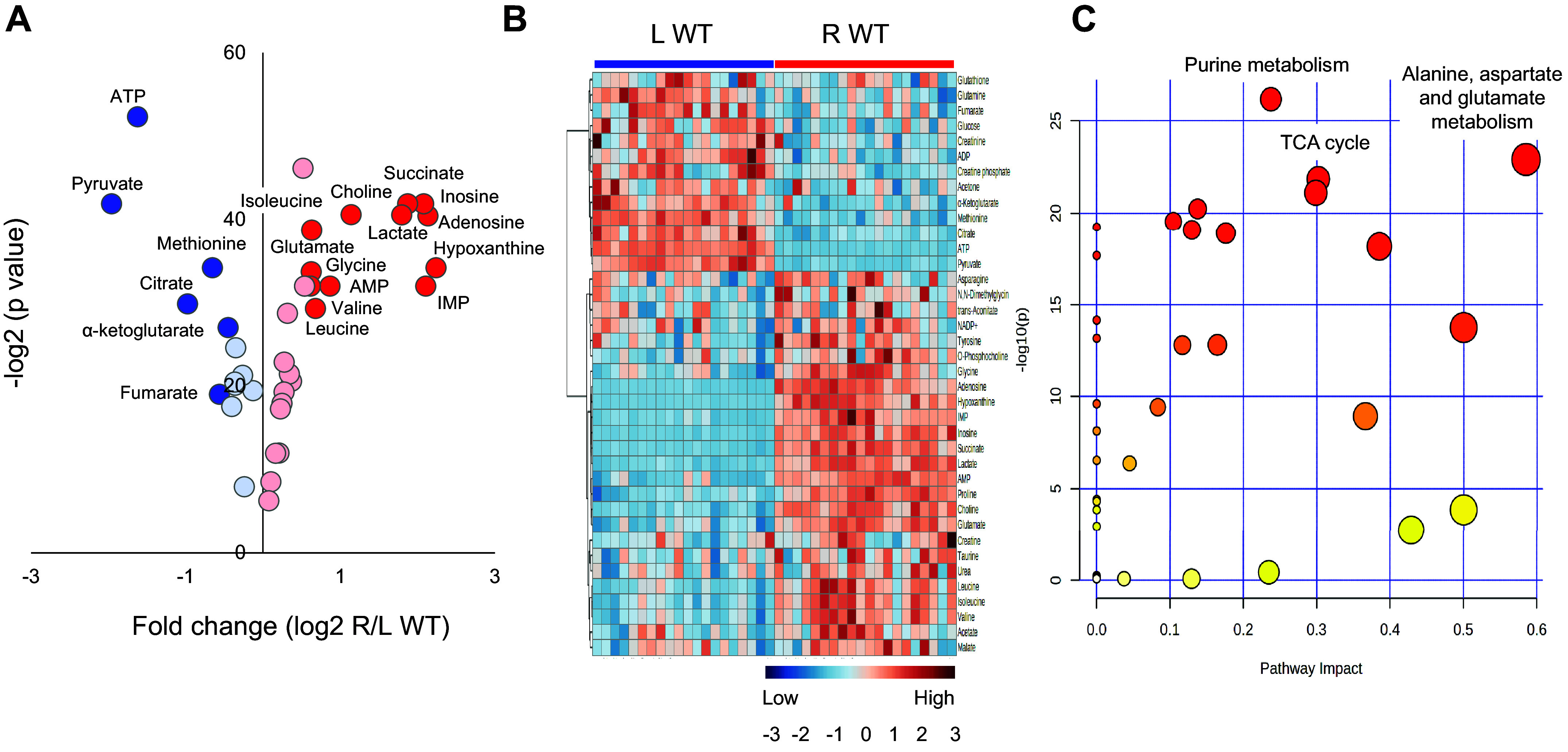
Volcano plot and heatmap of kidney tissue metabolites and pathway enrichment analysis in wild-type (WT) animals. A total of 49 metabolites were quantified in the left (L) WT clamp-frozen and right (R) WT snap-frozen kidneys. *A*: volcano plot analysis including 38 metabolites that presented a change (25 increased and 13 decreased), while 11 metabolites were stable (not shown). Thirteen metabolites, depicted in dark red, presented a fold change (FC) of ≥1.5. Contrarily, 6 metabolites, depicted in dark blue, presented a FC of ≤0.7. *B*: heatmap analysis of metabolites presenting a change between the left and right kidneys with a false discovery rate of ≤0.05. *C*: scatterplot depicting pathway enrichment analysis. The normalized topological measures of the altered metabolites in each pathway are summarized in pathway impact scores displayed on the *x*-axis. The enrichment analysis results −log_10_(*P*) values are displayed on the *y*-axis. The color gradients match the *y*-values of the data points, and the sizes of the data points are associated with their *x*-values. Pathways were considered significantly enriched if *P* < 0.05, impact > 0.1, and number of metabolite and number of metabolite hits in the pathway > 1.

Of 49 identified metabolites (Table 2), 38 differed between the two harvesting and freezing approaches (left vs. right kidney; FDR < 0.05). Twenty-five increased due to HF delay, but only 13 had a fold change of ≥1.5. Contrarily, 13 metabolites decreased, and only 6 presented a fold change of ≤0.7 (Fig. 3, *A* and *B*). Eleven metabolites were more stable with no change due to a brief HF delay. Most of the metabolic changes were consistent in males and females. However, a few changes were different among both sexes. All metabolic changes by sex are presented in Supplemental Table S6.

**Table 2.** Kidney metabolic changes with harvesting and freezing delay

Metabolite	FC WT	FC PKD	FDR LK Versus RK WT	FDR LK Versus RK PKD	FC WT Versus PKD
α-Ketoglutarate	**0.7**	**1.9**	**0.0000**	**0.0000**	**<0.0001**
Acetate	1.2	**1.5**	0.0000	**0.0000**	**<0.0001**
Acetone	0.8	NA	0.0041	NS	**0.0004**
Adenosine	**4.4**	**1.6**	**0.0000**	**0.0000**	**<0.0001**
ADP	0.8	0.9	0.0000	0.0000	NS
Alanine	NA	NA	NS	NS	NS
Allantoin	NA	**1.5**	NS	**0.0000**	**<0.0001**
AMP	**1.8**	**2.0**	**0.0000**	**0.0000**	NS
Asparagine	1.1	1.1	0.0027	0.0001	NS
Aspartate	NA	NA	NS	NS	NS
ATP	**0.3**	**0.4**	**0.0000**	**0.0000**	**0.0491**
Betaine	NA	NA	NS	NS	NS
Choline	**2.2**	**1.9**	**0.0000**	**0.0000**	NS
Citrate	**0.5**	**1.6**	**0.0000**	**0.0000**	**<0.0001**
Creatine	1.2	1.2	0.0000	0.0000	NS
Creatine phosphate	0.8	**0.6**	0.0000	**0.0000**	**<0.0001**
Creatinine	0.8	1.3	0.0000	0.0000	**<0.0001**
Dimethylamine	1.1	1.3	0.0131	0.0000	**<0.0001**
Formate	NA	NA	NS	NS	NS
Fumarate	**0.7**	NA	**0.0000**	NS	**<0.0001**
Glucose	0.8	0.9	0.0000	0.0000	**0.0001**
Glutamate	**1.5**	1.2	**0.0000**	0.0000	**<0.0001**
Glutamine	0.8	0.8	0.0000	0.0000	NS
Glutathione	0.9	1.3	0.0000	0.0000	**<0.0001**
Glycine	**1.5**	1.1	**0.0000**	0.0014	**<0.0001**
Hippurate	NA	1.4	NS	0.0000	**<0.0001**
Hypoxanthine	**4.7**	**3.7**	**0.0000**	**0.0000**	**0.0018**
IMP	**4.3**	**3.2**	**0.0000**	**0.0000**	**0.0041**
Inosine	**4.2**	**2.6**	**0.0000**	**0.0000**	**<0.0001**
Isoleucine	**1.5**	1.3	**0.0000**	0.0000	**<0.0001**
Lactate	**3.5**	**4.8**	**0.0000**	**0.0000**	**0.0004**
Leucine	**1.6**	1.4	**0.0000**	0.0000	**0.0048**
Malate	1.1	1.1	0.0003	0.0001	NS
Methionine	**0.6**	NA	**0.0000**	NS	**<0.0001**
*myo*-Inositol	NA	NA	NS	NS	NS
*N*,*N*-dimethylglycine	1.3	1.4	0.0000	0.0000	**0.0316**
NAD^+^	NA	NA	NS	NS	NS
NADP^+^	NA	NA	NS	NS	NS
*O*-phosphocholine	1.3	1.0	0.0000	0.0157	**<0.0001**
Proline	1.4	1.3	0.0000	0.0000	**<0.0001**
Pyruvate	**0.3**	NA	**0.0000**	NS	**<0.0001**
*sn*-Glycero-3-phosphocholine	NA	NA	NS	NS	NS
Succinate	**3.7**	**2.0**	**0.0000**	**0.0000**	**<0.0001**
Taurine	1.2	1.1	0.0000	0.0000	NS
*trans*-Aconitate	1.2	**1.5**	0.0002	**0.0000**	**<0.0001**
Trimethylamine	NA	NA	NS	NS	NS
Tyrosine	1.2	1.1	0.0000	0.0000	**<0.0001**
Urea	1.2	1.2	0.0000	0.0000	NS
Valine	**1.5**	1.2	**0.0000**	0.0000	**<0.0001**

FC, fold change; FDR, false discovery rate; LK, left kidney; NA, not applicable; NS, not statistically significant (FC is NA when NS); RK, right kidney; WT, wild type. Bold font indicates statistical significance.

The significant metabolic changes observed during a short HF delay were consistent with those caused by ischemic anoxia in the kidney. The most sensitive indicators of anoxia were the depletion of high-energy phosphate and the accumulation of purine nucleosides, including adenosine, inosine, and hypoxanthine, due to ATP catabolism. As expected, lactate concentration increased, while glucose, pyruvate, citrate, and α-ketoglutarate concentrations decreased. On the other hand, succinate and malate accumulated, likely due to reverse reactions of the tricarboxylic acid (TCA) cycle.

The overview of the pathway impact was analyzed using MetaboAnalyst 5.0. The top pathways affected by brief ischemic anoxia and freezing delay constitute purine metabolism, alanine, aspartate, and glutamate metabolism, and the TCA cycle (Fig. 3*C*). A detailed pathway analysis table, including all the identified pathways, is presented in Supplemental Table S7.

### Citrate and α-Ketoglutarate Accumulate With Ischemia in PKD

**Figure 4.**
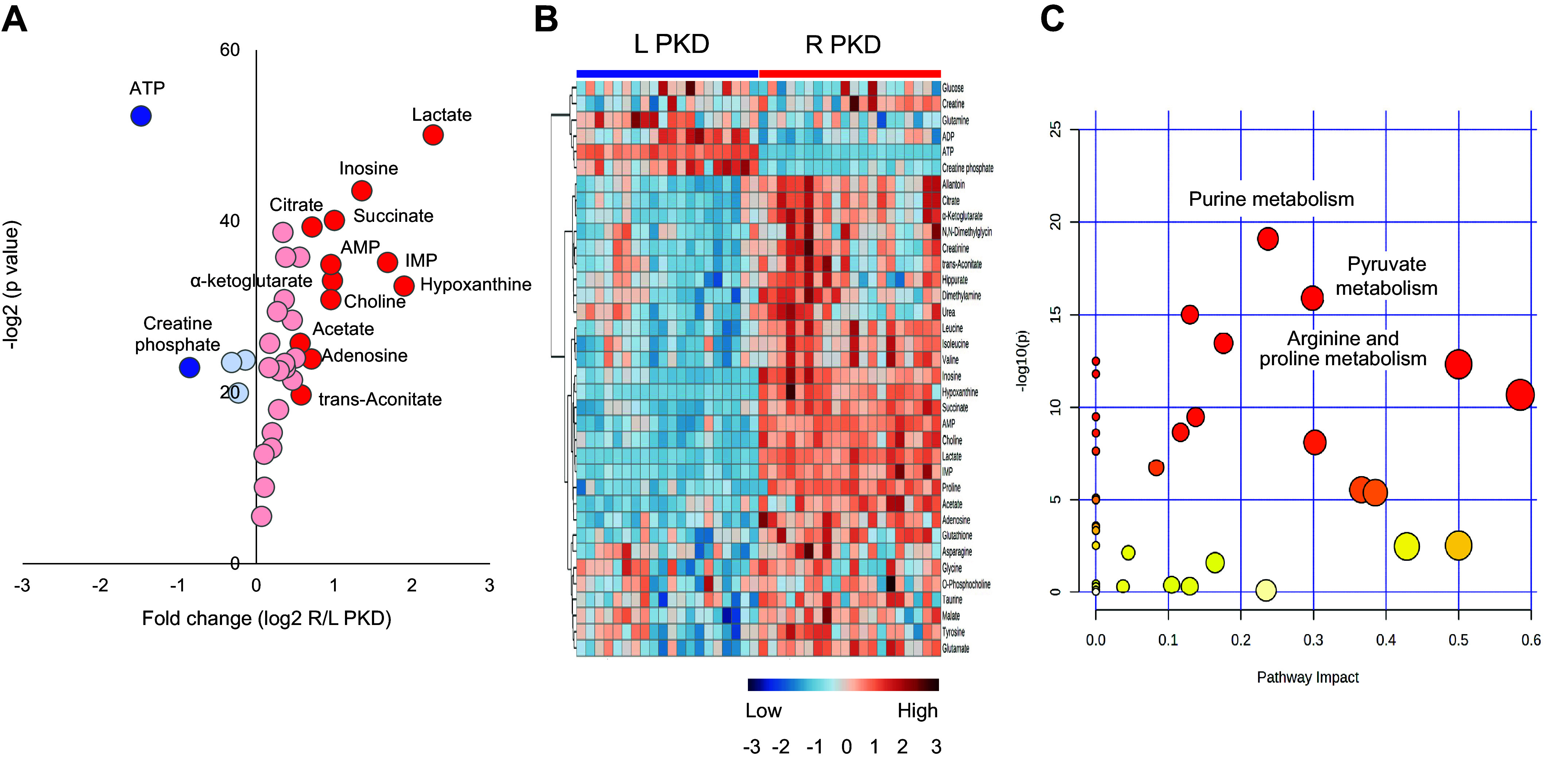
Volcano plot and heatmap of kidney tissue metabolites and pathway enrichment analysis in polycystic kidney disease (PKD). A total of 49 metabolites were quantified in the left (L) PKD clamp-frozen and right (R) PKD snap-frozen kidneys. *A*: volcano plot analysis including 37 metabolites that presented a change (31 increased and 5 decreased) while 13 metabolites were stable (not shown). Thirteen metabolites, depicted in dark red, presented a fold change (FC) of ≥1.5. Contrarily, only 2 metabolites in dark blue presented a FC of ≤0.7. *B*: heatmap analysis of metabolites presenting a change between the left and right kidneys with a false discovery rate of ≤0.05. *C*: scatterplot depicting pathway enrichment analysis. The normalized topological measures of the altered metabolites in each pathway are summarized in pathway impact scores displayed on the *x*-axis. The enrichment analysis results −log_10_(*P*) values are displayed on the *y*-axis. The color gradients match the *y*-values of the data points, and the sizes of the data points are associated with their *x*-values. Pathways were considered significantly enriched if *P* < 0.05, impact > 0.1, and number of metabolite and number of metabolite hits in the pathway > 1.

Similar to WT, 36 out of 49 metabolites differed between left and right kidneys (FDR < 0.05; Table 2). Thirty-one metabolites increased due to HF delay, but only 13 had a fold change of ≥1.5. Contrarily, only five metabolites decreased, and only two presented a fold change of ≤0.7 (Fig. 4, *A* and *B*).

As observed in WT, ATP and creatinine-p decreased, while purine nucleosides accumulated to a lesser extent. Similarly, glucose concentration decreased and lactate increased, but the rise in lactate was higher in PKD. Contrary to the expected decrease observed in WT, pyruvate, and fumarate concentrations did not change in PKD animals. Succinate accumulated as in WT but to a lesser extent. An unexpected finding was the accumulation of citrate and α-ketoglutarate during ischemia in PKD, likely resulting from a reductive carboxylation of glutamine-derived α-ketoglutarate to isocitrate and subsequent isomerization to citrate and consistent with prior reports of abnormal glutamine metabolism in PKD (Fig. 4, *A* and *B*) (19–21, 36).

Most of the metabolic changes were consistent in males and females. However, similar to WT, a few changes differed between the two sexes. All metabolic changes by sex are presented in Supplemental Table S8. Similarly to WT, purine metabolism, pyruvate metabolism, and arginine and proline metabolism were among the most impacted pathways (Fig. 4*C*). A detailed pathway analysis table, including all the identified pathways, is presented in Supplemental Table S9.

### Early PKD Presents With Increased Reactive Oxidation Products From Purine Catabolism and TCA Cycle Abnormalities

Previous studies have reported several metabolic abnormalities in PKD (6, 7, 17, 20, 37, 38). However, there have been some discrepancies among reports (7, 17, 39). To determine the closest to the in vivo metabolic changes in PKD, we compared the metabolic profile of the left kidney (without ischemic artifacts) from WT and PKD animals.

**Figure 5.**
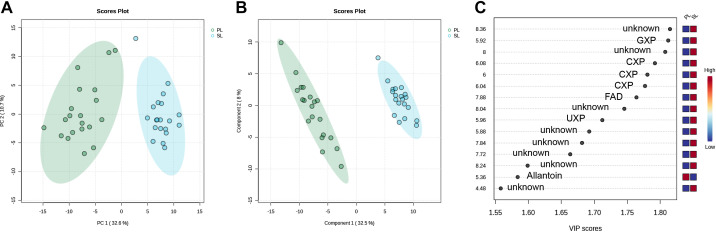
Proton nuclear magnetic resonance (^1^H-NMR) spectra analysis of wild-type (WT) and polycystic kidney disease (PKD) kidneys. *A*: unsupervised principal component (PC) analysis score plot based on binned spectra normalized by total area, discriminating between different genotypes (WT and PKD) from the extracts obtained from the left (nonischemic) kidneys. *B*: supervised partial least square discriminant analysis from the same animals. *C*: variable importance on projection (VIP) scores and metabolites related to bins with different concentration among the different genotypes (blue is low and red is high relative concentration). The explained variances along the PCs are shown in brackets. Shaded areas represent the 95% confidence regions. CXP, cytidine mono-, di-, and triphosphate; FAD, flavin adenine dinucleotide; GXP, guanosine mono-, di- and triphosphate; UXP, uridine mono-, di-, and triphosphate.

The unsupervised PCA of the binned WT and PKD left kidney spectra normalized to TA showed excellent separation between the two groups, with no overlap along PC1, explaining 32.6% of the total variance within the data and indicating that the kidney tissue metabolome differed significantly (Fig. 5*A*). PLS-DA and the resulting score plots of *component 1* versus *component 2* showing a clear separation are presented in Fig. 5*B*. Comparison between the WT and PKD kidneys without ischemic artifacts led to a model with good predictive values (*Q*^2^ = 0.953 and *R*^2^ = 0.987), which was further validated based on the permutation test with respect to the separation distance (*P* < 0.001). The computed VIP scores and overall coefficient scores from the PLS-DA for each bin are shown in Supplemental Table S10, and the top 15 features are shown in Fig. 5*C*. Metabolite assignment to the top 15 features indicated that bins associated with metabolites related to purine metabolism were some of the bins that most contributed to differentiating the two genotypes. Nevertheless, many spectral bins that resulted as significant features contributing to the model could not be attributed to a specific metabolite. Analyses using TSP-normalized spectra resulted in similar findings (Supplemental Fig. S4 and Supplemental Table S11).

**Figure 6.**
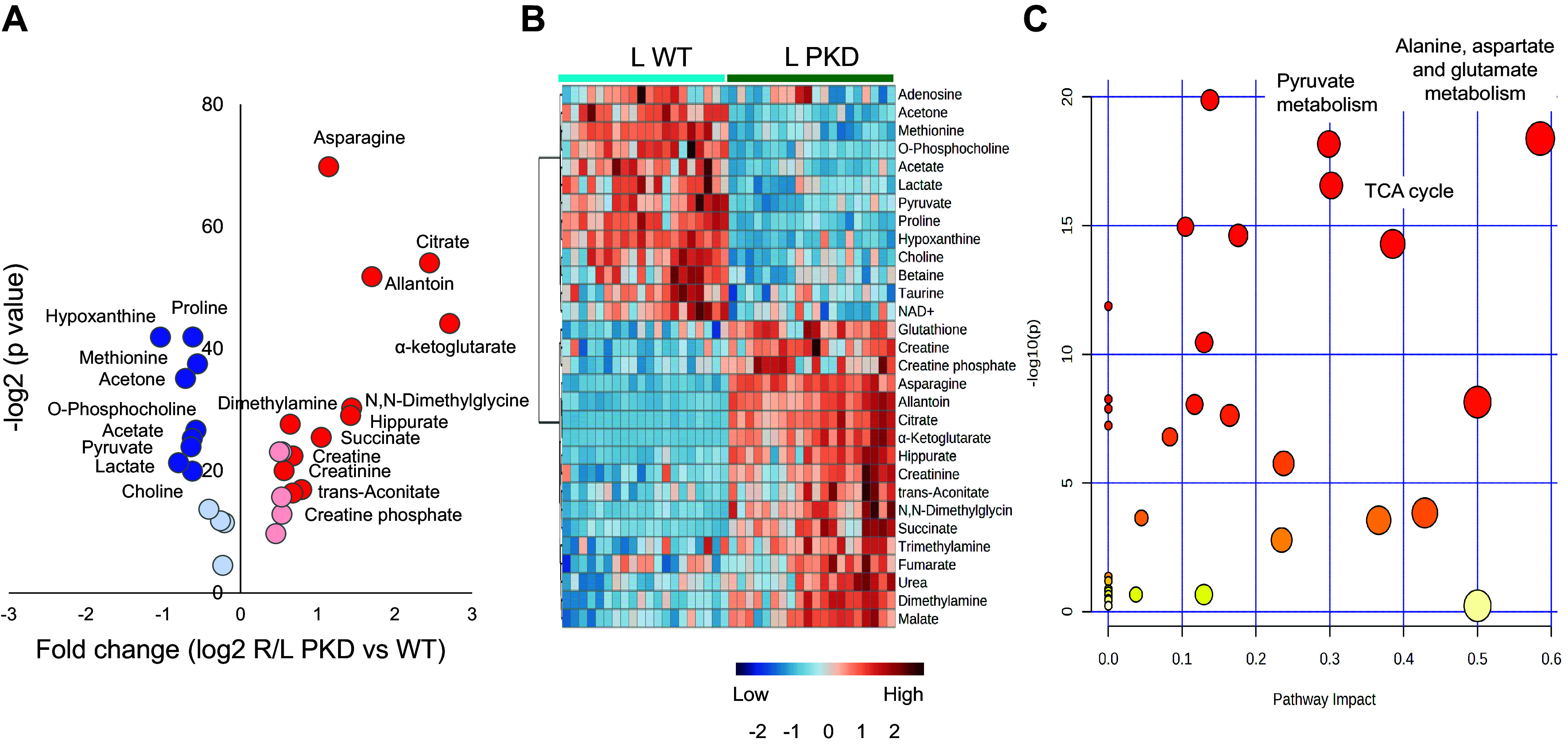
Volcano plot and heatmap of kidney tissue metabolites and pathway enrichment analysis in early polycystic kidney disease (PKD) vs. wild-type (WT). A total of 49 metabolites were quantified from the nonischemic kidneys in both genotypes. *A*: volcano plot analysis including 28 metabolites that were different among both genotypes (17 were higher and 13 were lower), while 19 metabolites were not different (not shown). Thirteen metabolites, depicted in red, presented a fold change (FC) of ≥1.5. Contrarily, 9 metabolites, depicted in blue, presented a FC of ≤0.7. *B*: heatmap analysis of metabolites that were different between genotypes (false discovery rate ≤ 0.05). *C*: scatterplot depicting pathway enrichment analysis. The normalized topological measures of the altered metabolites in each pathway are summarized in pathway impact scores displayed on the *x*-axis. The enrichment analysis results −log_10_(*P*) values are displayed on the *y*-axis. The color gradients match the *y*-values of the data points, and the sizes of the data points are associated with their *x*-values. Pathways were considered significantly enriched if *P* < 0.05, impact > 0.1, and number of metabolite and number of metabolite hits in the pathway > 1. L, left; R, right.

Metabolic comparison of the profiled spectra identified 30 metabolites with a FDR of <0.05 (Table 3). Seventeen metabolites were higher in PKD versus WT, and 12 had a fold change of ≥1.5. Contrarily, 13 metabolites were lower, and 9 presented a fold change of ≤0.7 (Fig. 6, *A* and *B*). Nineteen metabolites were not different between PKD and WT kidneys at the early stages of the disease. The most prominent differences observed in the clamp-frozen PKD kidneys were reflected by higher concentrations of α-ketoglutarate, citrate, and allantoin and lower hypoxanthine, lactate, and acetone (Fig. 6, *A* and *B*). To better understand the underlying differences in tissue lactate levels, we determine its concentration in blood and urine using ^1^H-NMR to examine its production and excretion. Plasma and urine lactate levels were not different between WT and PKD (not shown), further suggesting a decreased production in PKD rather than increased excretion.

**Table 3.** Metabolic changes in PKD

Metabolite	FC LK PKD Versus LK WT	FDR
α-Ketoglutarate	**6.5**	**0.0000**
Acetate	**0.6**	**0.0000**
Acetone	**0.6**	**0.0000**
Adenosine	0.9	0.0428
ADP	NA	NS
Alanine	NA	NS
Allantoin	**3.2**	**0.0000**
AMP	NA	NS
Asparagine	**2.2**	**0.0000**
Aspartate	NA	NS
ATP	NA	NS
Betaine	0.8	**0.0001**
Choline	**0.6**	**0.0000**
Citrate	**5.5**	**0.0000**
Creatine	**1.6**	**0.0000**
Creatine phosphate	**1.6**	**0.0000**
Creatinine	**1.5**	**0.0000**
Dimethylamine	**1.6**	**0.0000**
Formate	NA	NS
Fumarate	1.4	0.0012
Glucose	NA	NS
Glutamate	NA	NS
Glutamine	NA	NS
Glutathione	1.4	0.0000
Glycine	NA	NS
Hippurate	**2.7**	**0.0000**
Hypoxanthine	**0.5**	**0.0000**
IMP	NA	NS
Inosine	NA	NS
Isoleucine	NA	NS
Lactate	**0.6**	**0.0000**
Leucine	NA	NS
Malate	1.4	0.0000
Methionine	**0.7**	**0.0000**
*myo*-Inositol	NA	NS
*N,N*-dimethylglycine	**2.7**	**0.0000**
NAD^+^	0.9	0.0003
NADP^+^	NA	NS
*O*-phosphocholine	**0.7**	**0.0000**
Proline	**0.7**	**0.0000**
Pyruvate	**0.6**	**0.0000**
*sn*-Glycero-3-phosphocholine	NA	NS
Succinate	**2.1**	**0.0000**
Taurine	0.8	0.0003
*trans*-Aconitate	**1.7**	**0.0000**
Trimethylamine	1.3	0.0118
Tyrosine	NA	NS
Urea	1.4	0.0000
Valine	NA	NS

FC, fold change; LK, left kidney; NA, not applicable; NS, not statistically significant (FC is NS when NS); WT, wild type. Bold font indicates statistical significance.

Most of the metabolic changes were consistent in males and females. However, some changes were sex specific, and in some, a synergistic sex effect was observed. All metabolic changes by sex are presented in Supplemental Table S12. The overview of the impacted pathways identified alanine, aspartate and glutamate metabolism, pyruvate metabolism, and the TCA cycle as the top three differentially affected pathways (Fig. 6*C*). A detailed pathway analysis table, including all the identified pathways, is presented in Supplemental Table S13.

### Brief Harvesting-Freezing Delay Leads to False Positive and Negative Metabolite Differences Between WT and PKD Kidneys

A critical question is how much a HF delay may impact experimental results when comparing WT and PKD kidneys and how many differences may be confounded by ischemic artifacts. If the metabolic impact across both genotypes is similar, then an equal delay in harvesting and freezing would enable safe comparisons when clamp freezing is not feasible. Because we identified differences in the effect HF delay had in WT and PKD, we compared the kidney metabolic profile of the right kidney (subjected to HF delay) in WT and PKD. For these analyses, we considered the left kidney (clamp frozen) as the time of 0 min and the right kidney (delayed freezing) as the time of 8 min (average right kidney time in WT and PKD).

**Figure 7.**
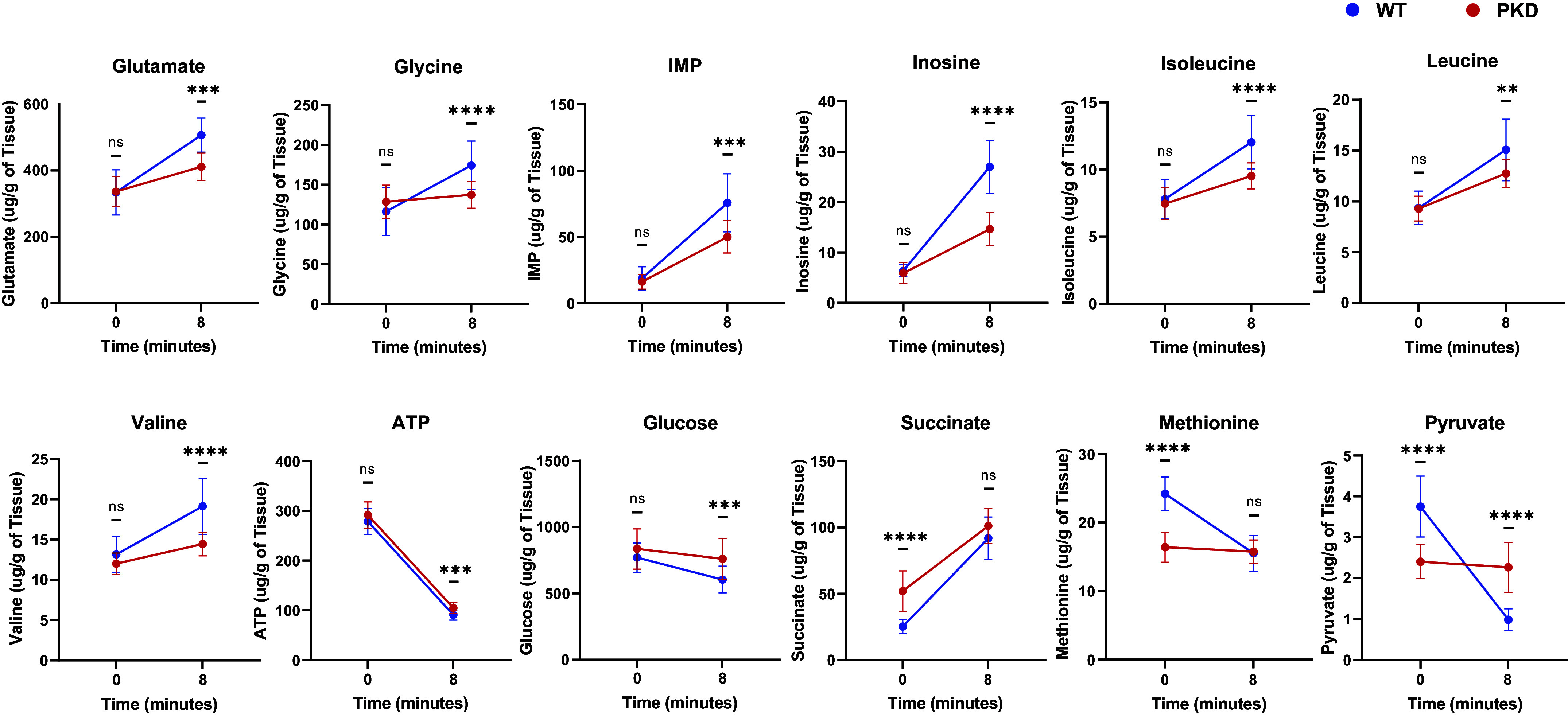
Brief harvesting-freezing delay led to false positive and negative metabolite differences between wild-type (WT) and polycystic kidney disease (PKD) kidneys. Nine metabolites (glutamate, glycine, IMP, inosine, isoleucine, leucine, valine, ATP, and glucose) did not differ at *time 0* in WT vs. PKD but became different at 8 min (false positive). Two metabolites (succinate and methionine) differed at *time 0*, but this difference was lost (false negative). Pyruvate was lower in PKD at *time 0* but became higher at 8 min compared to WT due to a different effect of delayed harvesting and freezing in both genotypes. Data are presented as means ± SD. *P* values between WT and PKD were calculated using Student’s *t* test with a Benjamini-Hochberg false discovery rate (FDR) post hoc correction to correct for multiple comparisons (FDR < 0.05 for statistical significance). All animals for the left (0 min) and right (8 min) kidneys were included in the analyses. **P < 0.01, ***P < 0.001, and ****P < 0.0001. ns, not significant.

Of the 40 metabolites that changed (Table 2) in WT or PKD with HF delay, 34 differed between WT and PKD after an 8-min delay (Supplemental Table S14). In 24 metabolites, the difference between WT and PKD remained in the same direction. However, nine metabolites were initially not different at *time 0* in WT versus PKD became different at 8 min (false positive). On the other hand, in two metabolites that were different at *time 0*, this difference was lost (false negative). Notably, in one metabolite (pyruvate), there was a difference at *times 0* and *8* between WT and PKD, but this difference was in the opposite direction with the HF delay (Fig. 7). Four metabolites remained not different between WT and PKD, despite being affected by HF delay due to having a similar change. Therefore, it is not safe to assume that equally delaying HF in WT and PKD kidneys controls for the effects of HF delay, ultimately leading to false negative and false positive genotypic differences.

## DISCUSSION

Prior studies determining ischemia’s effect on kidney metabolites’ content have been limited in the number of metabolites, were based on enzymatic assays, or applied to WT kidneys only (24, 33). Importantly, these studies have shown that some kidney metabolites can change in as little as 30 s. Although these findings are critical to the metabolic studies of the kidney, how they extend to the larger metabolome and affect a model of CKD has remained unaddressed. Our study using ^1^H-NMR in WT and PKD kidneys significantly extends prior studies and presents several novel findings. First, a brief HF delay significantly alters the metabolic profile of WT and PKD kidneys. Second, the metabolic changes observed with brief HF delay are consistent with those caused by ischemic anoxia in the WT kidney. Third, in contrast, while PKD kidneys present many expected ischemic changes, key notable differences were observed. Fourth, PKD kidneys collected minimizing ischemic artifacts and closely resembling the in vivo conditions present significant metabolic changes related to alanine, aspartate, and glutamate metabolism, pyruvate metabolism, and the TCA cycle. Fifth and finally, a brief HF delay leads to false positive and negative metabolite differences between WT and PKD kidneys.

The harvesting of tissues and organs is accompanied by anoxia, lack of substrate, and accumulation of metabolic waste, resulting from severing their blood supply. Certain organs, like the liver, use leftover oxygen and stored glycogen for continuous aerobic metabolism, but this process ends when oxygen is depleted. The mammalian kidney has no glycogen or glucose deposits and is thus wholly dependent on a continuous blood supply for glycolytic substrate and oxygen. The blood oxygen content in the kidney at the time of harvesting may be calculated to be ∼1.5 µmol/g fresh weight of tissue (40). This would provide an approximate 20-s oxygen supply for the kidney, consuming about 5 µmol O_2_/min/g (41). Therefore, metabolic changes related to the depletion of oxygen concentration are expected to occur in less than 30 s.

Nevertheless, in addition to the metabolic changes induced by ischemic anoxia, metabolite concentration can change due to rapid degradation and sensitivity to factors such as temperature, pH, etc. Therefore, any additional delays between organ harvest and complete cellular metabolism quenching contribute to metabolite profile alterations, producing results that might not represent the organ’s in vivo metabolic state. Prior research has established the relationship between tissue size and freezing time following immersion in refrigerant (33). For example, for a kidney sample of 500 mg, it would take 25 s for the innermost part to reach 0°C after immersion in N_2_. However, it has been previously shown that complete metabolic activity does not cease until cells reach the glass transition temperature of water below −137°C (42). This delay in temperature drop is attributed to the low thermal conductivity of the tissue and the Leidenfrost phenomenon (43). Using other refrigerants, such as isopentane, can reduce freezing time by overcoming the Leidenfrost phenomenon. Yet, this is less practical, and the freezing time remains longer than 15 s. A solution to overcoming the low thermal conductivity is to flatten the tissue between precooled aluminum blocks to increase the surface area exposed to the refrigerant, as initially developed in Wollenberger’s clamp-freezing approach (32).

Using our modified clamp-freezing technique, we minimized ischemia time to ∼7 s and freezing time to ∼5 s, resulting in a total time of <15 s from harvesting to reaching a temperature approximating that of N_2_ and providing a closer snapshot of the in vivo kidney metabolome. We further confirmed which metabolites are highly labile in the kidney and would require rapid inactivation of intracellular metabolism to eliminate biochemical artifacts and prevent degradation resulting from delayed kidney harvesting and freezing techniques. In addition, we determined a group of metabolites for which there may be a “grace period” during which metabolic processes continue unaltered after the interruption of blood supply.

In the WT kidneys, the most significant metabolic changes resulting from a short HF delay were consistent with those caused by ischemic anoxia, reflected by changes in purine metabolism. Additional notable changes were observed in the glycolytic and TCA cycle metabolites concentrations. The glucose, pyruvate, and TCA cycle metabolites’ levels decrease, and lactate accumulation is consistent with partial inhibition of glycolysis and TCA cycle during anoxia. The reduction in citrate, α-ketoglutarate, and fumarate levels was associated with increased malate and accumulation of succinate, similar to the ischemic response observed in the heart (44–51). Malate levels rise fourfold in myocardial ischemia (52), with a significant conversion of α-ketoglutarate to succinate (53–56). As a result, succinate exits the mitochondria and will inhibit hypoxia-inducible factor (HIF)-α prolyl hydroxylase, stabilizing and activating HIF-1α, initiating a hypoxic response (57, 58). Another significant change observed with ischemia was an increased choline concentration. The choline accumulation likely results from impaired cell membrane integrity and hydrolysis of choline-containing phospholipids and phospholipid Ch-derived intermediates (59). Most of the metabolite changes were similar in males and females.

Changes in purine metabolism, with depletion of high-energy phosphate and accumulating purine nucleosides, were also significant in PKD with harvesting and freezing delay. However, there are some notable differences between the resulting metabolic profile of ischemic anoxia in WT and PKD kidneys. As metabolic supplies and oxygen content decrease, pyruvate, citrate, and α-ketoglutarate levels decrease in the WT kidney, while succinate levels accumulate due to TCA cycle inhibition. In contrast, pyruvate levels did not change, while citrate and α-ketoglutarate levels increased and succinate accumulated to a lesser extent in PKD. This increase in citrate and α-ketoglutarate likely results from reductive carboxylation of glutamine-derived α-ketoglutarate and is consistent with increased glutamine utilization and prior reports of abnormal glutamine metabolism in PKD (19–21, 36). Interestingly, citrate and α-ketoglutarate were already higher in the clamp-frozen PKD kidney, further supporting abnormal in vivo glutamine metabolism in PKD. In highly proliferating cells, glutamine has been shown to be a primary anaplerotic substrate to replenish the TCA cycle intermediates, and the reductive carboxylation of glutamine carbon becomes the major pathway of citrate synthesis needed for lipid and cell membrane biosynthesis (60, 61).

Other notable differences in the clamp-frozen PKD kidneys were higher concentrations of allantoin and lower hypoxanthine, lactate, and acetone. A higher allantoin concentration results from increased purine catabolism, supported by the concomitant reduction in hypoxanthine. Hypoxanthine serves as a substrate for superoxide radical production by xanthine oxidase, leading to increased uric acid formation and its oxidation product, allantoin (62). Consequently, uric acid formation is used as an indicator of xanthine oxidase activity, and increased levels of allantoin are an accepted marker for in vivo free radical reactions, reflecting high levels of reactive oxygen species (ROS) (63–66). This is in line with our previous report of increased ROS production at the early stages of the disease in this model (67).

Higher rather than lower lactate levels might have been expected in PKD resulting from a Warburg effect and increased aerobic glycolysis or chronic hypoxia associated with cyst formation. Lower lactate levels may result from decreased production, increased excretion, or degradation. Although our studies are not labeling studies and prevent dynamic metabolic flux interpretation, the similar lactate concentrations observed in blood and urine between WT and PKD favor decreased production and rule out increased excretion (68, 69). While we cannot exclude lactate degradation as an explanation for the lower tissue concentration, lactate levels significantly increased with time in both WT and PKD, further distancing this possibility. Finally, we recently showed that the capillary index is not different in PCK rats versus WT at this early stage of disease (67), and therefore, oxygen supply is likely still adequate under continuous blood flow to the organ, decreasing the possibility of chronic hypoxia at this early stage. Therefore, the observed decreased levels most likely represent a decreased production at this early stage. Mechanistic, longitudinal studies are needed to determine the dynamics of the kidney metabolic abnormalities through different stages of disease.

Importantly, our study shows that a brief HF delay leads to significant changes in the kidney metabolome, and while many changes were similar in WT and PKD, the delay has different effects in some pathways for WT and PKD. The difference in this effect resulted in false-negative and false-positive differences between WT and PKD. It is also worth noting that while some metabolites changed in the same direction due to the harvesting and freezing delay, some changes were significantly different in magnitude between genotypes. An example of this was lactate. Levels of this metabolite were lower in the clamp-frozen PKD kidney, but their rise in PKD kidneys was significantly higher than in WT kidneys, reaching ∼400%. Although in our study the levels remained lower in PKD after ∼8 min of ex vivo ischemia, the difference between PKD and WT approximated. Notably, other studies in rodent liver have shown how a similar freezing delay impacts metabolite levels and alters TCA cycle intermediate ^13^C-isotopologue abundances and fractional enrichments of metabolites with differentially redox-regulated feeder pools (70). This further highlights the importance of reducing harvesting ischemia and immediate freezing to preserve accurate metabolite concentrations and ^13^C-isotopologue distributions and enrichments. These differences observed with harvesting and freezing delay may partly explain metabolic discrepancies observed between studies. Because of the biological relevance of glycolytic and TCA cycle metabolism in PKD and other kidney diseases, it emphasizes the importance of reporting and controlling sample handling to limit possible confounding artificial effects, not only in static metabolic studies but also to obtain ^13^C-tracing data that more accurately reflects the in vivo state.

Our study is limited by the inability to determine segment-specific metabolic changes to preserve the in vivo metabolic conditions and rapid termination of kidney metabolic activities; therefore, the results represent a global metabolic profile. Nevertheless, a close in vivo snapshot of the kidney may still provide valuable insights into the kidney metabolic microenvironment and reflect changes associated with the disease state. The administration of anesthetic agents to rodents to allow for organ harvesting preeuthanasia can affect the tissue metabolome. However, previous studies have shown that most variability related to metabolic changes is associated with how rapidly tissues can be harvested after anesthesia induction and becomes more critical in more lengthy procedures (71, 72). Furthermore, a study in rodents demonstrated that the metabolic profile of several organs overlapped between three different anesthetic methods, suggesting that the metabolome of tissues collected under anesthesia, in which respiration and blood circulation remain intact until the time of collection, are broadly similar regardless of what anesthetic agent is used (73). On the other hand, the ischemic anoxia induced by euthanasia is responsible for faster and more dramatic metabolite changes, and it is therefore recommended that rodent tissues intended for metabolomics studies be collected under anesthesia rather than posteuthanasia (73).

An alternative organ harvesting strategy suggested for other organs is performing saline perfusion before freezing (74). While this technique has the advantage of removing the majority of the blood, allowing for tissue metabolites to be distinguished from blood metabolites, it is still a lengthy procedure and would still be confounded by ischemic changes. Metabolic studies from blood and urine collected from the same animal at the time of euthanasia can aid in distinguishing organ versus biofluid metabolome. Consequently, we prefer to capture the combined tissue and residual blood and urine metabolome from anesthetized animals, as this is likely to reflect the true in vivo conditions more closely.

In summary, our study revealed profound metabolic changes resulting from harvesting and freezing delays in the WT and PKD kidneys. As a result, our laboratory routinely uses the modified clamp-freezing approach for metabolomics analyses in the kidney. Although we are limited to the global analysis of the kidney metabolic profile and residual blood and urine metabolome, our modified clamp-freezing technique likely reflects the actual in vivo conditions more closely. Taken together, the data obtained indicate that the quick clamp-freezing technique for kidney metabolomics provides a more accurate interpretation of the in vivo metabolic changes associated with the disease state.

## DATA AVAILABILITY

Data will be made available upon reasonable request. The raw binned data normalized to TA and TSP obtained from WT and PCK animals for left and right kidneys are available at https://doi.org/10.6084/m9.figshare.25705707.v1 and https://doi.org/10.6084/m9.figshare.25705647.v1.

## SUPPLEMENTAL DATA

10.6084/m9.figshare.26364439 Supplemental Figs. 1–4 and Supplemental Tables S1, S6, S7, S8, S9, S12, S13, and S14*A*: https://doi.org/10.6084/m9.figshare.26364439.

10.6084/m9.figshare.26364487 Supplemental Figs. 1–4 and Supplemental Tables S1, S6, S7, S8, S9, S12, S13, and S14*B*: https://doi.org/10.6084/m9.figshare.26364487.

## GRANTS

This work was supported by National Institute of Diabetes and Digestive and Kidney Grants DK128017 and DK118391 and Department of Defense-focused program funds (PR221810, to M.V.I.), nuSURF Program Grant DK101405, and the Pirnie Family Translational PKD Center.

## DISCLOSURES

No conflicts of interest, financial or otherwise, are declared by the authors.

## AUTHOR CONTRIBUTIONS

M.V.I. conceived and designed research; Y.A., I.M., S.Z., I.V., and M.V.I. performed experiments; Y.A., J.Z., I.V., and M.V.I. analyzed data; S.Z., I.V., S.M., and M.V.I. interpreted results of experiments; M.V.I. prepared figures; M.V.I. drafted manuscript; Y.A., I.M., J.Z., S.Z., I.V., P.D., S.M., and M.V.I. edited and revised manuscript; Y.A., I.M., J.Z., S.Z., I.V., P.D., S.M., and M.V.I. approved final version of manuscript.
